# Involuntary Treatment in Dementia Care at Home: Results From the Netherlands and Belgium

**DOI:** 10.1093/geroni/igaa057.2299

**Published:** 2020-12-16

**Authors:** Vincent Moermans, Angela Mengelers, Michel Bleijlevens, Hilde Verbeek, Frans Tan, Elizabeth Capezuti, Koen Milisen, Jan Hamers

**Affiliations:** 1 Maastricht University, Riemst, Limburg, Belgium; 2 Maastricht University, Maastricht, Limburg, Netherlands; 3 Care and Public Health Research Institute, Maastricht University, Maastricht, Limburg, Netherlands; 4 Hunter College of CUNY, New York, New York, United States; 5 KU Leuven, LEUVEN, Vlaams-Brabant, Belgium

## Abstract

Most PwD remain living at home. Due to complex care needs this can result in an increased risk for care provided against the wishes of the client and/or to which the client resists, referred to as involuntary treatment. This study explores the use and factors associated with involuntary treatment in PwD receiving home care in the Netherlands and Belgium. A secondary data analysis of two cross-sectional surveys (n=844 persons) showed that more than half of the PwD (51%) living at home received involuntary treatment (Belgium 68% and the Netherlands 45%). Non-consensual care (83%) was the most common, followed by psychotropic medication (41%) and physical restraints (18%). Involuntary treatment was associated with living alone, greater ADL dependency, lower cognitive ability, higher family caregiver burden and receiving home care in Belgium versus the Netherlands. In order to provide person-centered care, it is important to study ways to prevent involuntary treatment in PwD. Part of a symposium sponsored by Systems Research in Long-Term Care Interest Group.

